# In Vitro Antibacterial Potentials of *Cinnamomum verum* J. Presl (Cinnamon), *Citrus x aurantium* L. (Petitgrain) *Pelargonium graveolens* L’Hér (Rose Geranium), and *Thymus vulgaris* L. (Thyme) Essential Oil-Containing Ointments Against Canine Otitis Externa *Pseudomonas aeruginosa* Isolates

**DOI:** 10.3390/ph19101500

**Published:** 2026-09-22

**Authors:** Anita Seres-Steinbach, Ákos Jerzsele, Adrienn Mercedesz Veres, Viktória Balázs Lilla, Eszter Riethmüller, Boglárka Kiss, Krisztián Bányai, György Schneider

**Affiliations:** 1 Department of Medical Microbiology, Medical School, University of Pécs, H-7624 Pécs, Hungary; seres-steinbach.anita@edu.pte.hu; 2 Department of Pharmacology and Toxicology, University of Veterinary Medicine, H-1078 Budapest, Hungary; jerzsele.akos@univet.hu (Á.J.); veres.a.mercedesz@univet.hu (A.M.V.); krisztian.banyai@aok.pte.hu (K.B.); 3 National Laboratory of Infectious Animal Diseases, Antimicrobial Resistance, Veterinary Public Health and Food Chain Safety, University of Veterinary Medicine, H-1078 Budapest, Hungary; 4 Department of Pharmacognosy, Faculty of Pharmacy, University of Pécs, H-7624 Pécs, Hungary; balazs.viktoria@gytk.pte.hu; 5 Department of Pharmacognosy, Faculty of Pharmacy, Semmelweiss University, H-1092 Budapest, Hungary; riethmuller.eszter@semmelweis.hu; 6 Center for Pharmacology and Drug Research & Development, Semmelweis University, Üllői u. 26, H-1085 Budapest, Hungary; 7 Department of Clinical Centre, Institute of Clinical Pharmacy of Pécs, H-7624 Pécs, Hungary; 8 Department of Medical Biology, Medical School, University of Pécs, H-7624 Pécs, Hungary

**Keywords:** otitis externa, ointments, essential oils, *Pseudomonas aeruginosa*

## Abstract

**Background/Objectives**: Otitis externa is a common infection in dogs. The most important aetiological agent is *Pseudomonas aeruginosa*, and an increasing number of cases are proving difficult to treat due to this pathogen’s antibiotic resistance. **Methods**: We determined the composition of cinnamon (*Cinnamomum verum* J. Presl), petitgrain (*Citrus x aurantium* L.), rose geranium (*Pelargonium graveolens* L’Hér), and thyme (*Thymus vulgaris* L.) essential oils (EOs) using gas chromatography-mass spectrometry (GC-MS). We then used the bioautographic method to identify antibacterial components. Two-and-a-half- and five percent concentrations of the essential oils were added to four different ointment bases, such as hydrogelum methylcellulose, unguentum oleosum, unguentum stearini, and vaselinum cholesterinatum, and the in vitro antibacterial effects were tested on lawns of six *P. aeruginosa* isolates using the antibiotic-containing product Marbogen complex as a control. The keepability of the methylcellulose-based ointments was monitored at 4 °C and 27 °C for 12 months, with efficacy losses evaluated monthly. **Results**: Hydrogel methylcellulose (HM) was the most effective at supporting the therapeutic efficacy of each oil, reducing the bacterial count on the exposed area by 99.999%, 100%, 100%, and 100% in the case of 5% cinnamon, petitgrain, rose geranium, and thyme, respectively. EO-based HM ointments take effect within 15 min, with only slight improvement after 30, 45, and 60 min. Cinnamon was the most effective when applied in the HM matrix. In contrast to the HM and US gel matrices, the antibacterial supportive effects of *Unguentum oleosum* and *Vazelinum cholestrinatum* were drastically lower. The antimicrobial efficacy of essential oils in hydrogel formulation varied significantly depending on the type of oil (*p* < 0.001), its concentration (*p* < 0.001), the exposure time (*p* < 0.001), and the *P. aeruginosa* isolate used (*p* < 0.001). Cinnamon oil demonstrated the highest efficacy, achieving near-complete bacterial elimination across all isolates within 15 min. There was no significant difference between the 2.5% and 5% concentrations (EMM: 0.016 vs. 0.004; *p* = 0.904). **Conclusions**: Our results demonstrate that essential oil-based products can be effective alternatives to antibiotics when used in conjunction with an effective gel matrix.

## 1. Introduction

Otitis externa is one of the most common infections in pets, including dogs and cats. Recent studies have reported that otitis externa in dogs accounts for around 20% of dermatological consultations in general veterinary practice [1], and that nearly 76% of chronic otitis externa cases are associated with other dermatological conditions [2]. Symptoms of infection at this anatomical site include shaking the head, scratching the ears, a foul odor from the ears, redness, hair loss, signs of self-inflicted trauma to the pinna and surrounding area, facial discomfort or avoidance, and discharge, bleeding, and ulceration from the ear canal. Otitis externa can lead to infections affecting the middle or inner ear, which can have more serious consequences [2,3]. Primary factors that most commonly lead to otitis externa include allergies, tumors, endocrine disorders, and autoimmune diseases, while predisposing factors, such as moisture, increase the risk of developing otitis externa [2,3].

The most important aetiological agent of otitis externa is *Pseudomonas aeruginosa*, an opportunistic Gram-negative bacterium that is isolated from 20–60% [4,5,6,7] of the cases in companion animals [8]. This bacterium can survive across a wide range of environmental conditions and particularly prefers to colonize the ear canals of various mammalian hosts [9].

Managing *Pseudomonas* otitis externa involves identifying the cause of inflammation in the ear canal, cleaning and removing debris from the ear, treating any concurrent infections, and controlling inflammation. It also involves addressing or reversing any pathological changes in the ear [10]. Treatment for chronic infections can take several months, with local empirical therapy typically including aminoglycosides (mainly gentamicin and tobramycin), polymyxin B, and fluoroquinolones [7]. Recently, however, a growing body of evidence suggests that symptoms do not improve with treatment and that recurrences occur [11,12,13,14].

One reason for therapeutic failure is the emergence of antibiotic resistance among the aetiological agents of canine otitis externa. This effect specifically affects *P. aeruginosa*, which is known for its intrinsic, acquired, and adaptive resistance, which can block multiple drug classes. This fact raises the need to search for alternative therapeutic options.

Certain success rates have been achieved using non-antibiotic methods. These include local acidification using 2% acetic acid or vinegar [15,16], the use of antiseptic agents such as silver sulfadiazine or Tris-EDTA [17,18], and the application of bacteriophages [19,20]. These approaches help to overcome resistance by disrupting biofilms and lowering the pH to a level that is unfavorable to the causative agent. Another treatment option is the application of essential oils. Over the last two decades, a large body of research has demonstrated the various biological effects of essential oils, with several studies verifying the antibacterial properties of herbal extracts against different bacteria. One advantage of essential oils (EOs) over antibiotics is that they typically consist of several compounds—in certain cases, their number can reach 50 or more—and, as is becoming increasingly apparent today, each component can have different points of attack on or within the bacterial cell. In fact, some EOs resemble a mixture of antibiotics with different attack points [21,22]. This explains why resistance to them is rarely observed, making EO-based antibacterial therapies an ideal option for treating skin infections.

Although experimental evidence supports the direct application of EOs to eliminate *P. aeruginosa* and replace conventional antibiotics, translation of these results into a clinical context remains limited, with in vivo studies being scarce and controlled clinical trials completely lacking [23].

Some recent studies have investigated the supportive efficacies of Carbopol 934, Carbopol 940, or vaselin-, stearin-, hydrogel-, or natural-based products, such as beeswax, on the antibacterial efficacies of ointments and gels against different bacteria, including *Cutibacterium acnes*, *Staphylococcus aureus*, and *Staphylococcus epidermidis*, as well as *P. aeruginosa* [24,25,26,27,28]. These studies have also revealed that controlled release and biological activities depend on the base ointment applied and the chemical composition of the essential oils.

In a recent study, our group analyzed the antibacterial, anti-biofilm, and biofilm-degrading effects of 60 different essential oils on 40 different *P. aeruginosa* isolates from canine otitis externa. Our results revealed that cinnamon (*Cinnamomum verum* J.Presl), rose geranium (*Pelargonium graveolens* L’Hér), thyme (*Thymus vulgaris* L.), and petitgrain (*C. x aurantium*) were the most effective extracts in these tests. Based on these results, we focused our subsequent research on formulating potential therapeutics targeting *P. aeruginosa*. Therefore, the four most effective candidate essential oils (EOs) were mixed with four different ointment bases, and their in vitro antibacterial properties were tested. The following bases were used for this purpose, based on the Normales (FoNo) Editio VIII: Hydrogelum methylcellulose FoNo VIII; *Unguentum oleosum* FoNo VIII; Unguentum stearini FoNo VIII (Ung. stearini); and Vaselinum cholesterinatum FoNo VIII. (Vas. chol.) [24,25,26,27,28,29].

The aim of this study is to bridge the gap between research and practical application by suggesting a basic formulation for bioactive ointments containing essential oils (EOs) as active compounds. This would enable antibiotic-containing anti-otitis externa gels to be replaced, thereby reducing antibiotic usage and helping to curb the global spread of antibiotic resistance.

## 2. Results

### 2.1. Gas Chromatography

Based on GC-MS analysis, the components of cinnamon (*Cinnamomum verum* J. Presl) are linalool (8.8 min, 0.13%), trans-cinnamaldehyde (12.5 min, 94.41%), eugenol (13.8 min, 4.71%), β-caryophyllene (15.0 min, 0.29%), and, finally, caryophyllene oxide (17.8 min, 0.46%). The main component of cinnamon essential oil is trans-cinnamaldehyde (with a relative concentration of 94.41%) (Figure 1A).

Petitgrain *(Citrus x aurantium* L.) contained significantly more components. β-Pinene (0.45%) appeared at 6 min, followed by limonene (0.61%) at 7.2 min and linalool (11.36%) at 8.8 min. Other components included α-terpineol (10.8 min, 9.53%), γ-terpineol (10.9 min, 2.03%), methyl anthranilate (13.8 min, 65.95%), citronellyl acetate (14.6 min, 0.91%), β-caryophyllene (15.0 min, 5.70%), and caryophyllene oxide (17.8 min, 1.75%). Based on the analysis, the main component of the essential oil is methyl anthranilate (nearly 66%), followed by linalool (~11%) and α-terpineol (~9.5%) (Figure 1B).

In the case of rose geranium (*Pelargonium graveolens* L’Hér), we were also able to identify numerous components, listed in order of their retention times: α-pinene (5.1 min, 0.86%), phenethyl alcohol (9.1 min, 28.13%), menthone (10.0 min, 5.42%), benzyl acetate (10.1 min, 0.92%), isomenthone (10.2 min, 2.67%), β-citronellol (11.4 min, 22.8%), geraniol (11.9 min, 19.05%), phenethyl acetate (12 min, 3.55%), and benzyl benzoate (20.5 min, 1.05%). Rose geranium essential oil is a complex blend whose main components are phenethyl alcohol (~28%), β-citronellol (~23%), and geraniol (~19%) (Figure 1C).

In the case of thyme (*Thymus vulgaris* L.), the following essential oils were detected based on their retention times: p-cymene (7.1 min, 15.13%), linalool (8.7 min, 1.35%), borneol (10.2 min, 2.15%), terpinen-4-ol (10.5 min, 1.91%), thymol (12.7 min, 64.76%), carvacrol (12.9 min, 8.89%), and at 22.8 and 23.3 min, two different unknown compounds at 4.31% and 1.53%, respectively. The main component of the essential oil is thymol, accounting for 64.76% (Figure 1D).

### 2.2. Thin-Layer Chromatography-Based Bioautography

Direct bioautography based on thin-layer chromatography (TLC-DB) was performed to localize the antibacterial zones of cinnamon, petitgrain, rose geranium, and thyme essential oils against Pseudomonas aeruginosa isolates 8 and 37. Compound assignments were based on the chromatographic characteristics together with the GC-MS composition of the essential oils and literature data. Where available, these were further supported by TLC-DB analysis of commercially available reference compounds.

A prominent antibacterial zone was observed in cinnamon bark essential oil at Rf = 0.63. This was assigned to cinnamaldehyde based on the chromatographic profile and GC-MS composition, and this was further supported by analysis of the corresponding reference compound (Figure 2C,D(Cn)).

In the case of petitgrain EO, three chromatographic zones were found to inhibit the metabolism of *P. aeruginosa* (Figure 2C,D(Pg)). Antibacterial activity was detected at Rf values of 0.50, 0.30, and 0.21; however, these active zones could not be unequivocally assigned to individual constituents.

In the case of the rose geranium essential oil sample, two antibacterial zones were detected using the TLC-DB method (Figure 2C,D(Ro)). The antibacterial zone observed at Rf = 0.22 was tentatively associated with citronellol based on the GC-MS composition and chromatographic behavior, and the zone above this region at Rf = 0.32 was tentatively associated with geraniol. This assignment was additionally evaluated using the corresponding reference compound.

In the case of the thyme oil sample, a prominent antibacterial zone was observed at Rf = 0.56 and was associated with the thymol/carvacrol region based on the GC-MS composition and chromatographic behavior (Figure 2C,D(Th)). Additionally, an antibacterial zone was observed at Rf = 0.25 and was tentatively associated with linalool, based on the GC-MS composition and published chromatographic data [30].

### 2.3. Antibacterial Activity of Reference Essential Oil Constituents

To further support the assignment of the antibacterial zones observed in the essential oil chromatograms, the same experimental conditions were used to analyze commercially available reference compounds using TLC-DB. These experiments also enabled the antibacterial activity of the individual reference compounds to be evaluated directly against the two *P. aeruginosa* isolates.

Of the reference compounds investigated, cinnamaldehyde and thymol exhibited the most pronounced antibacterial activity, producing clearly visible inhibition zones at Rf = 0.56 and Rf = 0.58, respectively (Figure 3C,D(Ca,Th)). These active compounds generated well-defined clearing zones on the bioautographic plates, indicating strong inhibition of bacterial metabolic activity.

Eugenol also exhibited antibacterial activity, producing a detectable inhibition zone at Rf = 0.52, albeit less pronounced than that observed for thymol and cinnamaldehyde (Figure 3C,D(Eu)). In contrast, geraniol (Rf = 0.22) demonstrated only weak antibacterial activity under the applied experimental conditions (Figure 3C,D(Ge)).

Limonene, a non-oxygenated monoterpene hydrocarbon, exhibited little-to-no detectable antibacterial activity against *P. aeruginosa* in the TLC-DB assay (Figure 3C,D(Li)).

Overall, the results suggest that the antibacterial activity observed in the chromatographic fractions is predominantly associated with phenolic and oxygenated monoterpenes, whereas hydrocarbon monoterpenes, such as limonene, exhibit limited inhibitory effects.

### 2.4. Identification of the Ideal Ointment Base for Antibacterial Testing

Two-and-a-half and five percent concentrations of four tested essential oils (cinnamon, petitgrain, rose geranium, and thyme) were mixed into four different ointment bases to determine which bases best support the antibacterial properties of essential oils in a gel matrix. Of the ointment bases, methylcellulose hydrogel and unguent stearin reduced the bacterial count even in the absence of essential oil. No differences in bacterial count could be detected between the ointment matrix and the ointment-free control sample for U.o. and V.ch. Of the essential oils mixed into the ointment pads, cinnamon and petitgrain were the most effective, reducing the bacterial count by 90–100%, compared to a 2.5% essential oil control sample (Figure 4A). Increasing the essential oil concentration to 5% significantly increased the effectiveness. During the 30-min exposure period, the number of colonies dropped from 80,000 to between 156 and 0, depending on the essential oil and ointment base used (Figure 4B).

These results confirmed that methylcellulose was the most suitable ointment base, since it supported the antibacterial activity of the emulsified EOs most strongly. Therefore, this ointment base was used for further experiments.

### 2.5. Analysis of the Supportive Antibacterial Effect of Methylcellulose Gelmatrix

In addition to isolate 8, we expanded the range of isolates tested to include isolates 4, 10, 13, 33, and 37. Cinnamon (Figure 5A) was the most effective against all isolates. On the Raincloud plot, the flatness of the plots is clearly visible. The density function shows that a value of 1–1 stands out from the rest at the 15-min mark, and then flattens out as time progresses. In the case of petitgrain (Figure 5B), the impact of incubation time on reducing the pathogen count is evident. At the 15-min mark, several points are visible on the density function, but these decrease steadily until the 60-min mark is reached. Although rose geranium (Figure 5C) was able to significantly reduce the germ count from 80,000, the figure clearly shows that as many as 800 germs were still detectable at the 15-min mark. However, the boxplots clearly show that both the maximum and median decrease progressively over time. The boxplot for thyme (Figure 5D) also shows that the maximum value decreases over time. However, the reduction in the pathogen count is less pronounced than in the case of rose geranium, and outliers can still be seen in the density function. By contrast, no outliers are visible in the petitgrain plot by the 60-min mark.

Significant differences were observed between the isolates (*p* < 0.001; df = 5), and the ointment’s effectiveness also varies depending on the essential oil mixed with each base (*p* < 0.001; df = 4). The incubation time also plays an important role in the effectiveness of the ointment (*p* = 0.005; df = 3). The incubation times were 15, 30, 45, and 60 min (see Figure 5A–E).

Regarding the isolate results, significant differences were observed between several pairs: 8 and 37 (p_holm_ < 0.001), and 33 and 37 (p_holm_ < 0.001). There is also a significant difference between the results at the 15- and 60-min time points (p_holm_ = 0.004). Based on the average rankings (457.38, 426.89, 408.27, and 389.45), the measured value decreased continuously and evenly over time (from 15 to 60 min). Significant differences were also observed between cinnamon and the other essential oils. When petitgrain was compared, the *p*-value was <0.001, as it was for rose geranium and thyme.

A Generalized Linear Mixed Model (GLMM) was employed as the primary analytical model following a preliminary non-parametric Kruskal–Wallis comparative analysis due to the multifactorial and repeated-measures structure of the data. Time, concentration, and essential oil type were included as fixed-effect factors, while isolates and replicates were treated as random-effect grouping factors. Estimated marginal mean (EMM) values were determined on a log_10_(CFU + 1) transformed scale. A log_10_(CFU + 1) transformation was applied to stabilize the variance, as the data distribution was strongly right-skewed; lower values indicated a lower bacterial load. EMMs derived from the GLMM were then examined to evaluate the effects of the individual treatments in greater detail.

Based on the results, which aimed to reveal the precise mechanism of action of the investigated factors and their interactions on the antimicrobial efficacy, significant differences were identified among the essential oils tested (cinnamon, petitgrain, rose geranium, and thyme; df: 3, *p* < 0.001), the specific time points (15, 30, 45, and 60 min; df: 3, *p* < 0.001), and the concentrations (2.5% and 5%; df: 1, *p* < 0.001). There was no significant interaction (*p* = 0.052), nor between time and essential oil type (*p* = 0.215). However, a significant interaction was identified between concentration and essential oil type (*p* < 0.001), indicating that the effect of increasing the concentration depended on the type of essential oil used. The three-way interaction was not significant (*p* = 0.920). To examine the significant concentration × essential oil interaction in detail, we performed pairwise contrasts for each essential oil, comparing the estimated marginal means (EMMs) at the 2.5% and 5% concentrations by applying a Tukey correction. For cinnamon, near-complete inhibition was already achieved at the 2.5% concentration (EMM = 0.016), and increasing the concentration to 5% did not result in further significant reduction (EMM = 0.004; Tukey-adjusted *p* = 0.904). The difference between the 2.5% and 5% cinnamon concentrations was 0.012 (95% CI: −0.185–0.210). In contrast, increasing the petitgrain concentration from 2.5% to 5% resulted in a significant reduction (EMM: 0.445 vs. 0.121; difference = 0.323; 95% CI: 0.126–0.521; Tukey-adjusted *p* = 0.001). For rose geranium, increasing the concentration from 2.5% to 5% resulted in a significant decrease (EMM: 1.604 vs. 0.798; difference = 0.806; 95% CI: 0.608–1.003; Tukey-adjusted *p* < 0.001). For thyme, however, the EMM decreased from 0.653 to 0.309, representing a significant difference (difference = 0.344; 95% CI: 0.147–0.541; Tukey-adjusted *p* < 0.001).

Overall, therefore, increasing the concentration from 2.5% to 5% significantly reduced the *P. aeruginosa* count for petitgrain, rose geranium, and thyme. However, for cinnamon, this did not result in a further significant reduction.

The antibacterial effect of the essential oils, based on their average ranking, was as follows: cinnamon < petitgrain < thyme < rose geranium < positive control. Therefore, cinnamon was the most antibacterial and rose geranium the least.

Of the essential oils used, cinnamon was the most effective (Figure 5A), followed by petitgrain (Figure 5B), thyme (Figure 5D), and rose geranium (Figure 5C). In the case of cinnamon, the number of detectable colonies was zero after 15 min. In contrast, viable bacteria were still detectable after 15 min for petitgrain and thyme. For rose geranium, it is clear that the number of recoverable viable *P. aeruginosa* decreases over time.

We have previously observed how different strains respond to essential oils in biofilm eradication [8], and treatment with ointments also illustrates this well. Figure 5 illustrates the effectiveness with which certain essential oils reduce the number of germs on the different isolates tested. In the case of cinnamon (Figure 6A), there was no significant difference in effectiveness. The boxplots are flat, and the density function is negligible. Of the four essential oils tested, cinnamon oil was the most effective antimicrobial agent. Petitgrain (Figure 6B) was effective against most of the isolates used, although viable bacterial counts were still present at the 15th and 30th minutes. These were eliminated by the 45th and 60th minutes. The boxplots were flat for isolates 4, 10, 13, 33, and 37. Although there are outliers for isolate 33, the maximum of the boxplot is only higher in the case of isolate 8. The effect of rose geranium (Figure 6C) depended heavily on the isolate. For isolate 33, both the median and maximum values in the boxplot are higher than for the other isolates. The most sensitive isolates were 10 and 37. It showed good efficacy against isolates 4 and 13. Rose geranium proved to be the least effective essential oil in terms of inhibition.

In the case of thyme (Figure 6D), the plots are also flat. However, the density function shows that low colony counts are observed. Although there are occasional outliers (up to 200–400 colonies) for isolates 4, 8, 13, and 33, the medians and mean colony counts remain close to zero. This suggests that thyme is also a highly effective essential oil against the *P. aeruginosa* isolates studied.

### 2.6. Shelf Life and Statistical Analysis of Various Essential Oil-Based Ointments

*P. aeruginosa* isolates 8 and 37 were used in shelf life tests. The essential oil-based ointments were stored at 4 °C and 27 °C for 12 months. The antibiotic-containing product Marbogen complex was used as a control.

During the tests, the bacterial count of the tested *P. aeruginosa* isolates (8 and 37) was significantly reduced by all ointments containing essential oils. Rose geranium appeared to be the least effective against isolate No. 8. However, for isolate 37, the results of the shelf-life test for the essential oils paint a significantly different picture.

Cinnamon and petitgrain maintained the bacterial count at 0 log10 CFU/mL throughout the experiment. They demonstrated maximum inhibitory effects for 12 months. Thyme exhibited minor fluctuations with isolate 37 during the third, fourth, eighth, and ninth months. Rose geranium was the least effective of the essential oils tested. By the end of the experiment (12th month), all the essential oils had almost completely suppressed bacterial growth (see Figure 7A,B).

In general, the bacterial count in isolation 8 had been completely reduced by the 15th minute by a combination of cinnamon and the Marbogen complex, and this remained the case until the end of the 1-h measurement period. Petitgrain was still detectable at a minimal level after 15 min. Thyme showed a slower but steady decrease. Rose geranium had a much slower effect, but by the 60th minute it had also decreased. Rose geranium reduced the average CFU value for isolate 8 by 82.4% (rounded to 83%) between 15 and 60 min, and by 91% for isolate 37. Cinnamon was also effective immediately against isolate 37. At the 15-min mark, petitgrain was more effective against isolate 37 than isolate 8, suppressing the number of living cells by 1.4 to 0.1 (a 92.9% reduction for isolate 8), while the average bacterial count for isolate 37 decreased from 0.1 to 0.0 (a 100% reduction). Thyme and rose geranium were also more effective against isolate 37 at the 15-min mark (Figure 7C,D).

Before conducting the comparative analysis, the assumptions required for parametric testing were assessed. The Levene test showed that *p* < 0.001 and F = 10.832, indicating that the system is not homogeneous. Additionally, the Shapiro–Wilk test revealed a significant deviation from normality (W = 0.325, *p* < 0.001), suggesting that the normality assumption was violated. Since both assumptions were violated, a non-parametric Kruskal–Wallis test was performed. The results of the Kruskal–Wallis test showed a significant difference in the bacterial counts among the different isolates (df = 1, H = 65.78, *p* < 0.001). There were also significant differences among the essential oils, the positive control, and the Marbogen complex used (df = 5, H = 2489.2, *p* < 0.001). There was no significant difference in shelf life between ointments stored at room temperature and those stored in the refrigerator (4 °C) (H = 0.072; df = 1; *p* = 0.789). Significant differences were observed between incubation times (H = 54.495, df = 3, *p* < 0.001). We monitored the shelf life of the ointments for 12 months and observed a significant difference in the bacterial counts over time (H = 111.791, df = 11, *p* < 0.001).

Dunn’s post hoc analysis showed that the positive control exhibited a significant difference compared to all essential oils (*p* < 0.001). In the tests, the Marbogen complex was used as the antibiotic control. There was no significant difference compared to cinnamon (*p* = 0.809); however, all other combinations showed significant differences. For example, cinnamon versus petitgrain/rose geranium/thyme; Marbogen complex versus all other essential oils except cinnamon; petitgrain versus rose geranium/thyme; and finally, rose geranium versus thyme. Cinnamon and the Marbogen complex had similar effects.

Significant differences in incubation times were revealed (*p* < 0.001): between 15 and 30, 15 and 45, 15 and 60, and 30 and 60. However, no significant differences were found between 30 and 45 (*p* = 0.085) or 45 and 60 (*p* = 0.090).

Overall, cinnamon exhibited the lowest estimated bacterial counts, followed by petitgrain.

To investigate the factors influencing the antimicrobial efficacy of the essential oils further, a Generalized Linear Mixed Model (GLMM) was subsequently applied to the data obtained for the four essential oil treatments (cinnamon, petitgrain, rose geranium, and thyme). This analysis focused specifically on the relative efficacy of the essential oils and the effect of essential oil concentration, while accounting for the repeated-measures structure of the experiment. The ointment-base control, the untreated control, and the antibiotic reference treatment were excluded from this EO-specific model because the concentration variable refers specifically to the essential oil concentration in the ointment and therefore cannot be meaningfully applied to the control and reference formulations.

The GLMM analysis revealed that the following factors had significant effects on the bacterial count: concentration (df = 1, *p* < 0.001), essential oil type used in the ointment (df = 3, *p* < 0.001), incubation time (df = 3, *p* < 0.001), and month of the study (df = 11, *p* < 0.001) had significant effects on the bacterial count. In contrast, no significant difference was observed between the storage temperatures tested (df = 1, *p* = 0.080). There were significant interactions between concentration and essential oil type (χ^2^ = 230.171, *p* < 0.001), essential oil type and incubation time (χ^2^ = 101.652, *p* < 0.001), essential oil type and storage temperature (χ^2^ = 85.940, *p* < 0.001), and essential oil type and study month (χ^2^ = 483.214, *p* < 0.001). These findings suggest that the impact of concentration, incubation time, storage temperature, and storage duration on bacterial counts varies depending on the essential oil used.

The estimated marginal means indicated the lowest bacterial counts for cinnamon and petitgrain, followed by thyme and rose geranium. For cinnamon, the estimated marginal mean decreased from 0.125 (standard error (SE) = 0.121, 95% confidence interval (CI): −0.112 to 0.361) at a concentration of 2.5% to −0.112 (SE = 0.121, 95% CI: −0.349 to 0.125) at a concentration of 5%. For petitgrain, the corresponding EMMs were 0.210 (SE = 0.121, 95% CI: −0.027 to 0.447) and −0.027 (SE = 0.121, 95% CI: −0.263 to 0.210), respectively. For rose geranium, the EMM decreased from 0.977 (SE = 0.121, 95% CI: 0.740 to 1.214) at 2.5% to 0.740 (SE = 0.121, 95% CI: 0.504 to 0.977) at 5%. For thyme, the decrease was from 0.461 (SE = 0.121, 95% CI: 0.224 to 0.697) to 0.224 (SE = 0.121, 95% CI: −0.013 to 0.461). Thus, the estimated marginal means were consistently lower at the 5% concentration than at 2.5% for all four essential oils. However, this reduction was statistically significant only for rose geranium and thyme, with no significant concentration effect detected for cinnamon or petitgrain. As the essential oil type × concentration interaction was significant (χ^2^ = 230.171, df = 3, *p* < 0.001), pairwise comparisons were subsequently performed to determine whether increasing the concentration from 2.5% to 5% resulted in a statistically significant reduction for each essential oil.

As the interaction between essential oil type and concentration was significant (*p* < 0.001), subsequent pairwise comparisons were performed to determine whether increasing the concentration from 2.5% to 5% resulted in a significant reduction in bacterial counts within each essential oil. The effect of concentration differed among the essential oils. For cinnamon, the difference between the 2.5% and 5% concentrations was not significant (EMM = 0.012, SE = 0.032, 95% CI: −0.050 to 0.075, z = 0.393, pholm = 0.694). Similarly, the difference for petitgrain did not reach statistical significance (EMM = 0.055, SE = 0.032, 95% CI: −0.007 to 0.118, z = 1.746, pholm = 0.081). However, increasing the concentration significantly reduced bacterial counts for rose geranium (EMM = 0.639, SE = 0.125, 95% CI: 0.394–0.885, z = 5.099, pholm < 0.001) and thyme (EMM = 0.252, SE = 0.032, 95% CI: 0.190–0.315, z = 7.949, pholm < 0.001).

Over time, the value of the investigated response variable decreased gradually and consistently. Based on the estimated marginal means, the highest value was observed at the 15-min time point (EMM = 0.500; SE = 0.120; 95% CI: 0.264; 0.736), followed by the 30-min time point (EMM = 0.359; SE = 0.120; 95% CI: 0.123; 0.594), and then the 45-min time point (EMM = 0.263; SE = 0.120; 95% CI: 0.027; 0.499). The lowest value was measured at the 60-min time point (EMM = 0.178; SE = 0.120; 95% CI: −0.058; 0.413). Pairwise contrasts confirmed that the response at 60 min was significantly lower than at 15 min (difference = 0.322; SE = 0.022; z = 3.803; *p* < 0.001), 30 min (difference = 0.181; SE = 0.022; z = 8.062; *p* < 0.001), and 45 min (difference = 0.085; SE = 0.022; z = 3.803; *p* < 0.001). Therefore, the response variable decreased significantly from 15 to 60 min, with the strongest difference observed between the 15- and 60-min time points. Overall, the results indicate a consistent time-dependent decline in the investigated response variable.

Examining the effectiveness of each essential oil at 4 °C and 27 °C reveals that, in the case of cinnamon, there was no significant difference between the two temperatures (z = −0.393; pholm = 0.694). Conversely, for petitgrain, we see that it was less effective at 4 °C than at 27 °C (z = −3.662; pholm < 0.001). The opposite effect was observed for rose geranium: it was more effective at 4 °C than at 27 °C (z = 6.399; pholm < 0.001). Thyme was also more effective at higher temperatures (z = −5.809; pholm < 0.001). In summary, petitgrain and thyme were less effective at 4 °C, while rose geranium was more effective at 4 °C than at 27 °C. There was no significant difference for cinnamon.

Examining the change in the effectiveness of the essential oils over time, we observe that cinnamon exhibited remarkable stability, with estimate values of 0.036 and 0.028 following log10CFU + 1 transformation. These values were not significantly different from zero (pholm = 0.785 and pholm = 0.830). In the pairwise comparisons, none of the paired months tested showed a significant difference from the other 11 months tested when compared to the first month.

Petitgrain exhibited a higher bacterial count in the first month (EMM = 0.263) than cinnamon. However, its EMM decreased to 0.032 by month 12, suggesting that its antibacterial activity remained consistent and increased over the study period, as predicted by the model estimates.

In the case of rose geranium, we see that the essential oil proved to be more effective with time. The EMM value decreased from 1.423 to 0.095 between month 1 and 12. This difference was statistically significant (SE = 0.078, z = 17.071, pholm < 0.001), indicating a significantly lower bacterial count after 12 months than at the start of the storage period. Thus, not only was the antibacterial activity of rose geranium maintained, but it also increased significantly over the storage period. Unlike rose geranium, thyme proved to be more effective from month 1 onwards, as the EMM value of 0.585 was lower than that of rose geranium. This EMM value decreased to 0.096 by month 12. This difference was also statistically significant (SE = 0.078, z = 6.283, *p* < 0.001). The four essential oils exhibited distinct temporal patterns. Cinnamon had the most stable effect, with no significant change in effectiveness over the year (pholm = 1.000). Cinnamon exhibited the greatest temporal stability, with no significant variations in bacterial counts between month 1 and subsequent months. In the case of petitgrain, rose geranium, and thyme, higher EMM values were observed in the first months, indicating lower effectiveness compared to cinnamon. However, as time progressed, their effectiveness did not deteriorate, although the EMM decreased in all cases.

Overall, cinnamon was found to reduce bacterial counts the most. Higher concentrations of essential oil resulted in lower bacterial counts, although this was only demonstrated for rose geranium and thyme. Incubation time also affected efficacy.

## 3. Discussion

*Pseudomonas aeruginosa* is the most significant aetiological agent of otitis externa in dogs. Although recent studies have summarized the importance of other agents, such as *Malassezia pachydermatis*, *Staphylococcus pseudintermedius*, *Proteus mirabilis*, *Escherichia coli*, *Streptococcus canis*, *Corynebacterium* spp., *Enterococcus* spp., *Klebsiella* spp., and *Enterobacter* spp. [7], *P. aeruginosa* is still considered the most important agent due to its increasing resistance to antibiotics and the ability to develop characteristic biofilms. This is why our study focused on this microorganism. Another reason is that this bacterium is a zoonotic agent that can colonize not only dogs, but also humans, causing diseases such as otitis externa, eye inflammation, wound infections, and generalized systemic infections with serious consequences [31].

This study involved six *P. aeruginosa* isolates selected from a recently established collection of 40 *P. aeruginosa* isolates from canine otitis externa [8]. The bacterial isolates (4, 8, 10, 13, 33, and 37) were selected for the experiments due to their significant biofilm-forming capacities. Biofilms were considered because they can significantly limit the efficacy of essential oils. However, biofilms cannot hinder the antibacterial effects of cinnamon, petitgrain, rose geranium, and thyme, which is why they were chosen for this study [8]. Since all of the tested *P. aeruginosa* isolates were sensitive to these four EOs, we considered it justified to investigate these essential oils in more detail as potential anti-otitis externa agents that could replace antibiotics. Based on the One Health Concept, this latter aspect is an important one because, to slow down the spread of antibiotic resistance, we should replace antibiotics where possible [32,33]. Quality control of the four EOs using Gas-Chromatography revealed that they exhibited typical compound compositions for the relevant plant EOs (Figure 1). The cinnamon EO contained 94.41% *trans*-cinnamaldehyde; the petitgrain sample contained 65.95% methyl anthranilate; the rose geranium EO contained phenethyl alcohol (28.13%), β-citronellol (22.84%), and geraniol (19.05%); and the thyme oil contained 64.76% thymol. These values are consistent with recent data. The antimicrobial effectiveness of essential oils is known to depend closely on the chemical structure, functional groups, and concentrations of their major active components. Essential oils dominated by aldehydes and phenols generally exhibit the highest potency, followed by those rich in terpene alcohols, while ester-dominated matrices typically show moderate to weak activity [34]. Trans-cinnamaldehyde is an unsaturated aldehyde; methyl-anthranilate is an aromatic ester; citronellol and geraniol are monoterpene alcohols; and thymol is a phenolic monoterpene [34,35,36,37]. Although the above-mentioned major compounds are pivotal in the antibacterial effect, it should be noted that the cumulative effect of minor compounds can significantly influence the antibacterial effect of major compounds. This was observed in the case of limonene, which alone showed no antibacterial effect (Figure 3C,D(Li)), whereas petitgrain exhibited a characteristic white spot at its Rf 50 value, indicating antibacterial activity (Figure 2C,D(Pg)). This indicates a synergistic effect, which is consistent with a recent finding [38].

Finally, it is worth mentioning that the composition of an EO can be influenced by many factors, such as the geographical location of cultivation, the harvesting period and season, general weather conditions, and the processing method used to obtain the extract [39]. However, recent studies have shown that soil and water characteristics also influence composition [40]. Knowledge of these factors is important since it enables the composition of compounds to be increased and optimized for therapeutic purposes.

To address the quality concerns, a direct bioautography method based on thin-layer chromatography was used to evaluate and confirm the presence of the antibacterial compounds in these four EOs. The GC and the TLC-BA results are consistent with recent findings, which revealed cinnamaldehyde (in cinnamon), limonene (in petitgrain), geraniol (in rose geranium), and thymol (in thyme) to be antibacterial components. These findings were confirmed at the level of the major compounds by ordering new samples and performing TLC-BA on them.

It is also worth noting that the antibacterial effect of an EO depends not only on the components present in larger quantities (major compounds) but also on those present in a ratio of about 8–10%, as was recently reported [41]. This is why the synergistic effect of compounds should not be overlooked. Based on the research results from the past two decades, we know that the most important groups of compounds possessing antibacterial activities are phenolic compounds, aldehydes, oxygenated monoterpenes, terpene alcohols, terpenic ketones, and esters [42].

Thymol, cinnamaldehyde, and eugenol all contain polar functional groups. This makes them active and able to readily interact with components of the bacterial membrane. This may explain why they were dissolved easily in the hydroxypropyl methylcellulose gel matrix, were released relatively easily, and demonstrated their antibacterial properties in HM when applied in vitro on an agar plate. In contrast, the lower antibacterial activity of these essential oils (EOs) in *Unguentum oleosum* and *Vazelinum cholestrinatum* ointment bases (Figure 4) could be explained by the greater retentive power of these more hydrophobic matrices regarding the antibacterial components, meaning they were less effective at eliminating the present bacteria. The 90% antibacterial activity observed after 15 min of exposure (Figure 5) makes the HM hydrogel ointment base an ideal candidate for future applications [43]. The mild but clear antibacterial effect observed in Hydrogelum methylcellulose and Unguentum stearini (Figure 4) stems from their physical properties [44]. Methylcellulose binds water molecules in its matrix, thereby lowering the amount of freely available water around the bacterial cell [45]. Meanwhile, the oily stearic acid interacts with the lipid bilayers of the bacterial cell, destabilizing the cellular membrane and causing structural leakage [46]. In contrast, the hydrophobic ointment bases *Vaselinum cholesterinatum* and *Unguentum oleosum* do not have such antibacterial properties; however, their retention effects are greater since most of the compounds are hydrophobic.

Earlier studies have also documented that due to their hydrophilic and hydrophobic features, cellulose-based ointments, such as hydroxypropylmethylcellulose (HPMC), sodium carboxymethylcellulose (NaCMC), and methyl cellulose (MC), are effective gel bases for the effective release of therapeutic agents such as the first-generation antihistamine chlorpheniramine maleate [47]. This molecule contains both a hydrophobic aromatic ring structure and a hydrophilic, charged amino side chain [48]. Recent articles have reported similar results when EOs were applied in ointments [24,49]. Due to their excellent physical and chemical properties, hydrogels are considered to be potential materials for developing wound dressings with antibacterial activity [50,51]. These dressings are ideal for flat skin areas; however, the inner surface of the ear is not typically a body part where these dressings can be applied. However, hydrogels applied as a spray or ointment with the correct consistency can be ideal for otitis externa, where reaching the jagged affected areas of the auricle is crucial [52]. However, the essential oil evaporates quickly, whereas the gel matrix retains it, ensuring a prolonged biological effect [53,54].

In this study, we evaluated whether the effectiveness of the four tested ointments was significantly impaired during one year of storage (Figure 7). This is a crucial practical consideration influenced by the stability of the antibacterial components and the nature of the gel matrix applied [55,56,57,58,59,60,61].

## 4. Materials and Methods

### 4.1. Bacterium Strains

This study used different *P. aeruginosa* isolates (4, 8, 10, 13, 33, 37), which were previously isolated from dogs with clinical signs of otitis externa [8]. The isolates were cultured using MacConkey and selective cetrimide agar (Sharlau Chemie, Sentmenat, Spain). Gram staining and oxidase assays were performed on the cultures, and the bacteria were identified using matrix-assisted laser desorption/ionisation time-of-flight mass spectrometry (MALDI-TOF MS) (Bruker, Billerica, MA, USA). All isolates were stored at −80 °C in Mueller–Hinton medium supplemented with 20% glycerol and subcultured onto 5% sheep blood agar plates prior to experimentation [8].

### 4.2. Essential Oils

Based on our recent results [8], four essential oils were used in this study: cinnamon (*Cinnamomum verum* J.Presl), petitgrain (*Citrus x aurantium* L.), rose geranium (*Pelargonium graveolens* L’Hér), and thyme (*Thymus vulgaris* L’Hér). Cinnamon EO was obtained from bark sticks. Petitgrain and rose geranium EOs were derived from the leaves, young branches, and twigs, while the thyme essential oil was obtained from the above-ground parts of the plant (the flowering shoots, leaves, and stems). All EOs were extracted by steam distillation and purchased from A.G. Industries (Noida, UP, India). Compound compositions of the EOs were tested before the experiments using gas chromatography.

### 4.3. Gas Chromatography

For the analysis, 1 µL of essential oil was introduced into a 20 mL HS vial fitted with a silicone/PTFE septum. Static headspace solid-phase microextraction (sHS-SPME) was conducted with a CTC Combi PAL autosampler (CTC Analytics AG, Zwingen, Switzerland). The vial was first equilibrated at 100 °C for 5 min, after which a 65 µm StableFlex DVB/CAR/PDMS fiber (Supelco, Bellefonte, PA, USA) was inserted into the vial headspace. The extraction step was performed at 100 °C for 20 min. Subsequently, the fiber was thermally desorbed in the GC injector at 250 °C for 1 min, using a split injection with a 1:90 split ratio. Following desorption, the SPME fiber was cleaned and reconditioned under a flow of high-purity nitrogen at 250 °C for 15 min.

Chromatographic separation and mass spectrometric detection were carried out using an Agilent 6890N/5973N GC-MSD system (Santa Clara, CA, USA) fitted with a Supelco SLB-5MS capillary column (30 m × 250 µm × 0.25 µm). The oven was initially maintained at 60 °C for 3 min and was then heated to 250 °C at 8 °C/min, with the final temperature maintained for an additional 1 min. High-purity helium (6.0) served as the carrier gas at a constant flow of 1.0 mL/min (37 cm/s). Mass spectra were acquired with a quadrupole mass-selective detector operating in electron ionization mode at 70 eV. Full-scan acquisition was performed over the 41–500 amu range at a rate of 3.2 scans/s. Chromatographic data were evaluated using MSD ChemStation D.02.00.275 software (Agilent). Compound identification was based on comparison of retention times and mass spectra with those of reference standards and entries in the NIST 2.0 library. The relative composition of the samples was determined by area normalization.

### 4.4. Identification of Antibacterial Zones by Thin-Layer Chromatography-Based Bioautography

In order to reveal the antibacterially active compounds of the tested essential oils, Thin-Layer Chromatography-Based Bioautography (TLC-DB) was used, which has three major steps: separation of the compounds with TLC, visualization of the separated compounds with vanillin–sulphuric acid staining, and postchromatographic detection of antibacterial compounds. For these investigations, the *Pseudomonas aeruginosa* strain 8 (MDR), 37 was used [62].

#### 4.4.1. Thin-Layer Chromatography

Chromatographic separation was carried out on 5 × 10 cm silica gel 60 F254 aluminum-backed TLC plates (Merck, Darmstadt, Germany). The EOs were dissolved in absolute ethanol to prepare stock solutions (concentration: 150 mg/mL for cinnamon and 250 mg/mL for other essential oils), and precise volumes (1, 2, and 3 µL) were applied to the TLC plates using Finnpipette pipettes (Merck, Darmstadt, Germany). Following sample application, the chromatograms were developed using an optimized mobile phase. For EO separation, a solvent system of toluene/ethyl acetate (95:5) was employed, while dichloromethane was specifically used for cinnamon bark EO. Ascending chromatography was performed in a pre-saturated twin-trough chamber (Camag, Muttenz, Switzerland). All chromatographic procedures were conducted at ambient temperature (22 °C).

#### 4.4.2. Vanillin–Sulphuric Acid Staining

After development, the plates were dried at 90 °C for 5 min to ensure complete solvent removal. Separated constituents were visualized using ethanolic vanillin–sulfuric acid reagent, according to Wagner and Bladt [30]. Tentative identification was based on Rf values and the characteristic coloration of reference compounds. Additionally, visualization was performed under UV light at 254 nm. It is important to emphasize that TLC plates intended for bioautography were not subjected to vanillin–sulfuric acid staining, as this treatment interferes with the microbiological analysis steps involved in TLC-DB.

#### 4.4.3. Post-Chromatographic Detection of Antibacterial Compounds

Following chromatographic separation, the TLC plates were exposed to a *Pseudomonas aeruginosa* suspension to assess antibacterial activity. To ensure uniform bacterial adhesion and distribution across the plate surface, the chromatographic layers were immersed in 100 mL of the bacterial suspension. Subsequently, the plates were transferred into a low-wall horizontal incubation chamber (dimensions: 20 × 14.5 × 5 cm) and incubated at 37 °C for 2 h under controlled conditions.

For the detection of antibacterial zones, the TLC plates were briefly immersed (5 s) in an aqueous solution of 3-(4,5-dimethylthiazol-2-yl)-2,5-diphenyltetrazolium bromide (MTT; 0.05 g dissolved in 85 mL) (Sigma-Aldrich Ltd., Darmstadt, Germany), followed by incubation at 37 °C for 24 h. The tetrazolium salt MTT is metabolically reduced by viable bacterial cells to an insoluble formazan product, yielding a bluish-violet coloration. The appearance of distinct white zones against this colored background indicated areas of suppressed dehydrogenase activity, attributed to the antibacterial properties of the investigated essential oils or their major constituents.

#### 4.4.4. Antibacterial Compound Confirmation

Essential oil compounds with characteristic antibacterial features and determined by their Rf values were confirmed by the previously described thin-layer chromatography-based bioautography (TLC-DB) method. Compounds for confirmation such as limonene, eugenol, geraniol, thymol, and cinnamaldehyde were bought from Merck Co. (Darmstadt, Germany).

For chromatographic mobilization on the 60 F254 TLC plates, references were dissolved in absolute ethanol to prepare stock solutions with a concentration of 20 mg/mL. Aliquots of 0.5 and 1 µL were applied onto the plates corresponding to 0.01 and 0.02 mg per spot deposited on the chromatographic layer.

Development of the chromatograms was performed in a toluene–ethyl acetate (95:5, *v*/*v*) solvent as described above, and after development, the plates were dried at 90 °C for 5 min; spots were visualized with ethanolic vanillin–sulfuric acid reagent and UV light at 254 nm. The other plates were immersed in 100 mL of *P. aeruginosa* suspension and developed with MTT using the same conditions as described above.

### 4.5. Ointment Bases, Essential Oil Containing Ointments

Four different ointment bases, with different basic characteristics, were used in this study (Table 1). The basic ointments were prepared at the Institute of Clinical Pharmacy at the University of Pécs according to the Formulae Normales (FoNo) Editio VIII, such as Hydrogelum methylcellulose FoNo VIII, *Unguentum oleosum* FoNo VIII. (Ung. oleoso), Unguentum stearini FoNo VIII. (Ung. stearin), and Vaselinum cholesterinatum FoNo VIII. (Vasel. cholest.). The aim of this part of the study was to find an ideal ointment base whose composition best supports the topical antibacterial features and ensures good keepability of the antimicrobial ointment. Ointments were prepared as previously described [63].

Different concentrations of the four essential oils were emulsified in the ointment bases and left to rest for at least 24 h at 8 °C. The final oil concentrations were 2.5% and 5%.

### 4.6. Antibacterial Testing of the Essential Oil Containing Ointments on P. aeruginosa

Antibacterial testing of the ointments was conducted in two sections according to the previously described experimental setup with minor modifications [63].

Before the experiments, the optical densities (OD) of the bacteria were synchronized at 620 nm (OD_620_) to 0.2 (~10^8^ CFU/mL) in LB medium. In each experiment, the CFUs were verified by 100× serial dilutions. For this, 10 µL from the dilution steps were dropped onto the surface of LB agar medium, and the drop was drained from one edge to the other edge of the agar. Plates were incubated overnight at 37 °C. The next day, the colonies were counted, multiplied back by the dilution ratios, and colony-forming units were expressed for 1 mL (CFU/mL). The limit of detection was 100 CFU/mL.

To test the antibacterial efficacies of the ointments, 100 microliters of standardized bacterial suspensions were spread on the LB agar with the help of a glass rod to form a continuous lawn. After drying, approximately 50 µL volumes of the appropriate ointments were transferred onto the surfaces of the agar media containing the relevant lawns of test bacteria. The transferred test ointments covered about 1 cm^2^ of the bacterial lawn. One group of ointments, consisting of simple ointment bases without essential oils, served as negative ointment controls. Ointment bases with emulsified EOs contained 2.5% and 5% cinnamon, rose geranium, petitgrain, and thyme EO.

After 30 min of incubation at room temperature, 3 × 3 mm^2^ agar cubes were cut from the treated area and control spots (uncovered bacterium lawn area) with a sterile spatula. These agar cubes were transferred into 300 µL PBS and vigorously vortexed for 3 × 20 s. Subsequent CFU determinations were performed by serial dilutions from the 300 µL suspensions.

In vitro efficacy tests were repeated three times, and the results were calculated based on the bacterial count comparison of the control area of a 3 × 3 mm unit area and the bacterial count recovered from the treated area after 30 min of incubation.

After determining which ointment base best supported the manifestation of the antibacterial effect of the essential oils against the *P. aeruginosa* isolate 8, the experiments were repeated only with methylcellulose using 6 different isolates (4, 8, 10, 13, 33, 37) and multiple sampling times such as 15, 30, 45, and 60 min, in order to obtain a view of antibacterial kinetics.

Antibacterial tests were performed in three replicates. The limit of detection (LOD) of this experiment was 100 CFU/mL.

### 4.7. Shelf Life Experiments

After preparing 20 mL 5% methylcellulose hydrogel ointment bases, essential oil emulsifications were made from cinnamon, petitgrain, rose geranium, and thyme in separate 2 mL volume tubes. End concentrations were 2.5% and 5%. The tubes were stored at 4 °C and 23 °C, and their antibacterial efficacies were tested every month for a one-year period of time. For this one-year shelf life test, the *P. aeruginosa* isolates 8 and 37 were used, and tests were performed as described above. Shelf life experiments were performed in 3 individual replicates.

### 4.8. Statistical Analysis

Statistical analyses of the three parallel measurements were performed using JASP software (version 0.18.3.0). The differences in bacterial counts in response to the applied ointment bases, essential oil concentrations, essential oil types, and incubation times were initially evaluated using the Kruskal–Wallis test. A value of *p* < 0.05 was considered significant. For the subsequent GLMM analysis, bacterial counts were analyzed on a log10(CFU + 1)-transformed scale to reduce the effect of the strongly right-skewed distribution and stabilize the variance. Essential oils, concentrations, exposure time, storage duration, storage temperatures, and their interaction terms were included as fixed-effect factors, whereas isolates and replicates were modeled as random grouping factors. The estimated marginal means (EMMs) reported below therefore represent model estimates on the transformed scale. For Figure 5 and Figure 6, however, the original, non-transformed bacterial counts were retained to facilitate biological interpretation and provide a more intuitive visualization of the data distributions.

## 5. Conclusions

The in vitro antibacterial efficacies of cinnamon, petitgrain, rose geranium, and thyme, along with methylcellulose hydrogel, against the otitis externa *Pseudomonas aeruginosa* isolates suggest that natural components have the potential to replace antibiotics for the treatment of auriculum infection in dogs. After our promising preliminary results, in vivo tests or studies have to be performed to evaluate efficacy in real conditions. Further studies must be performed to specify the exact ointment compositions taking into account all aspects of commercial ointment formulations.

## Figures and Tables

**Figure 1:**
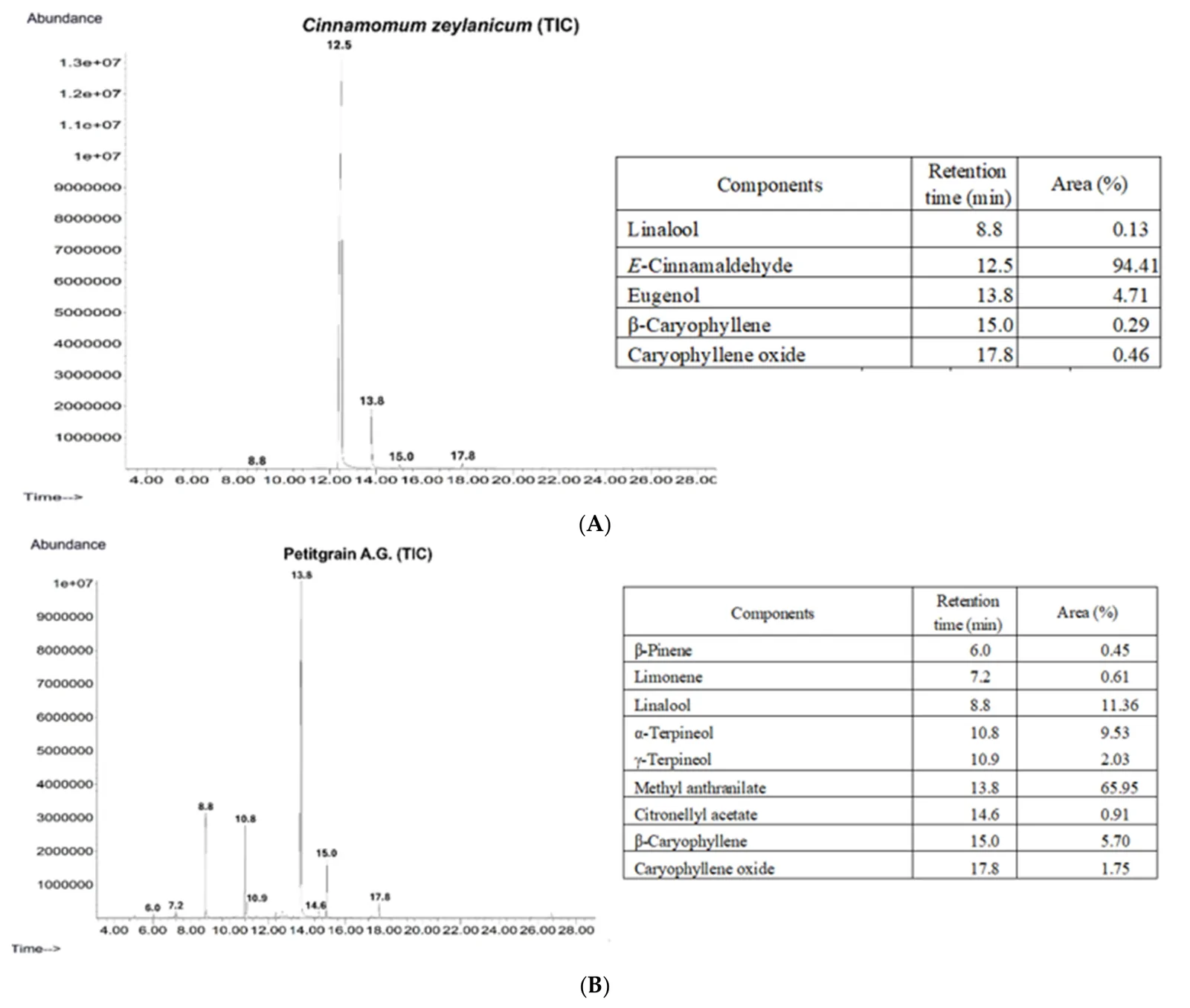

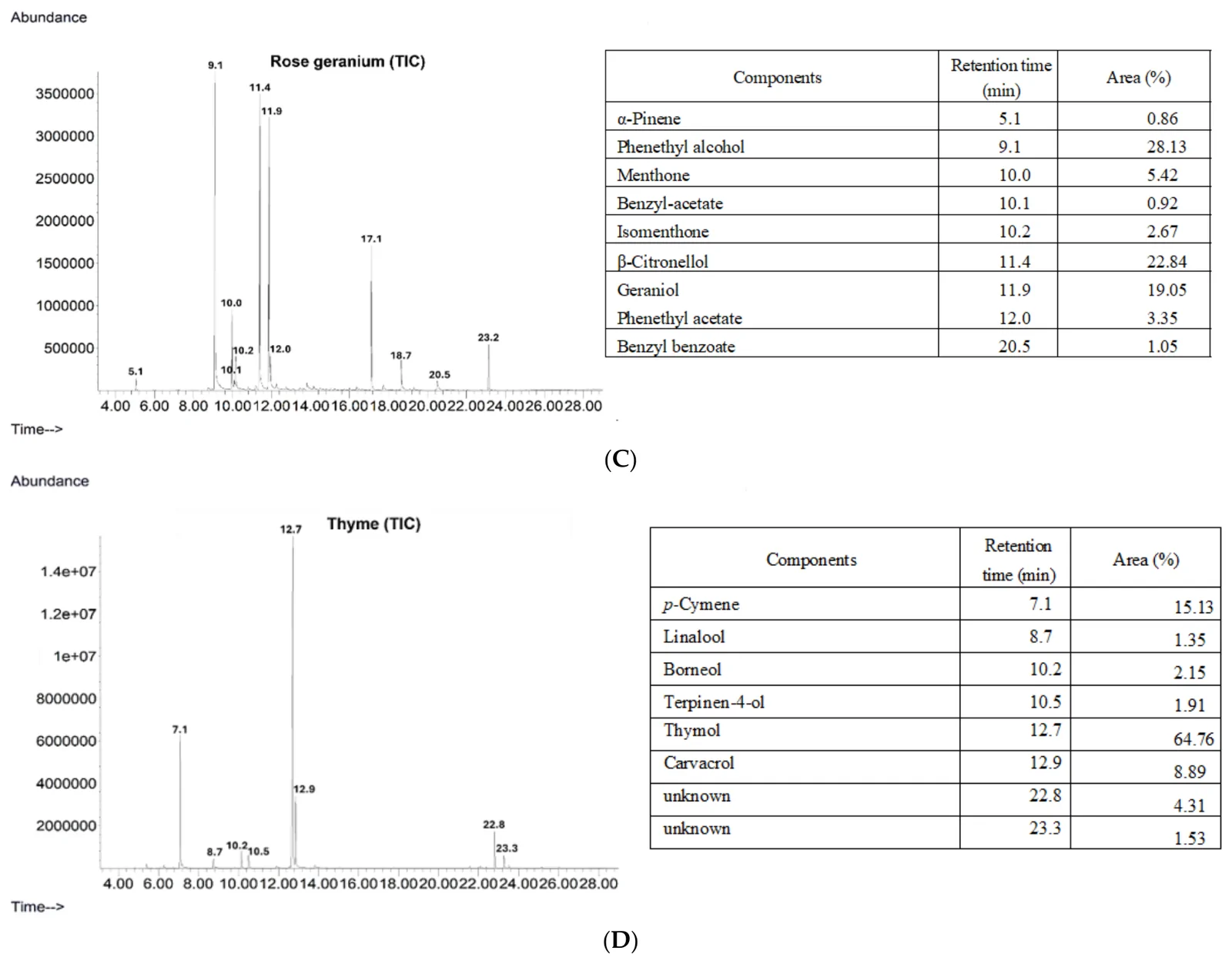
GC-MS results for the essential oils of cinnamon (**A**), petitgrain (**B**), rose geranium (**C**), and thyme (**D**).

**Figure 2:**
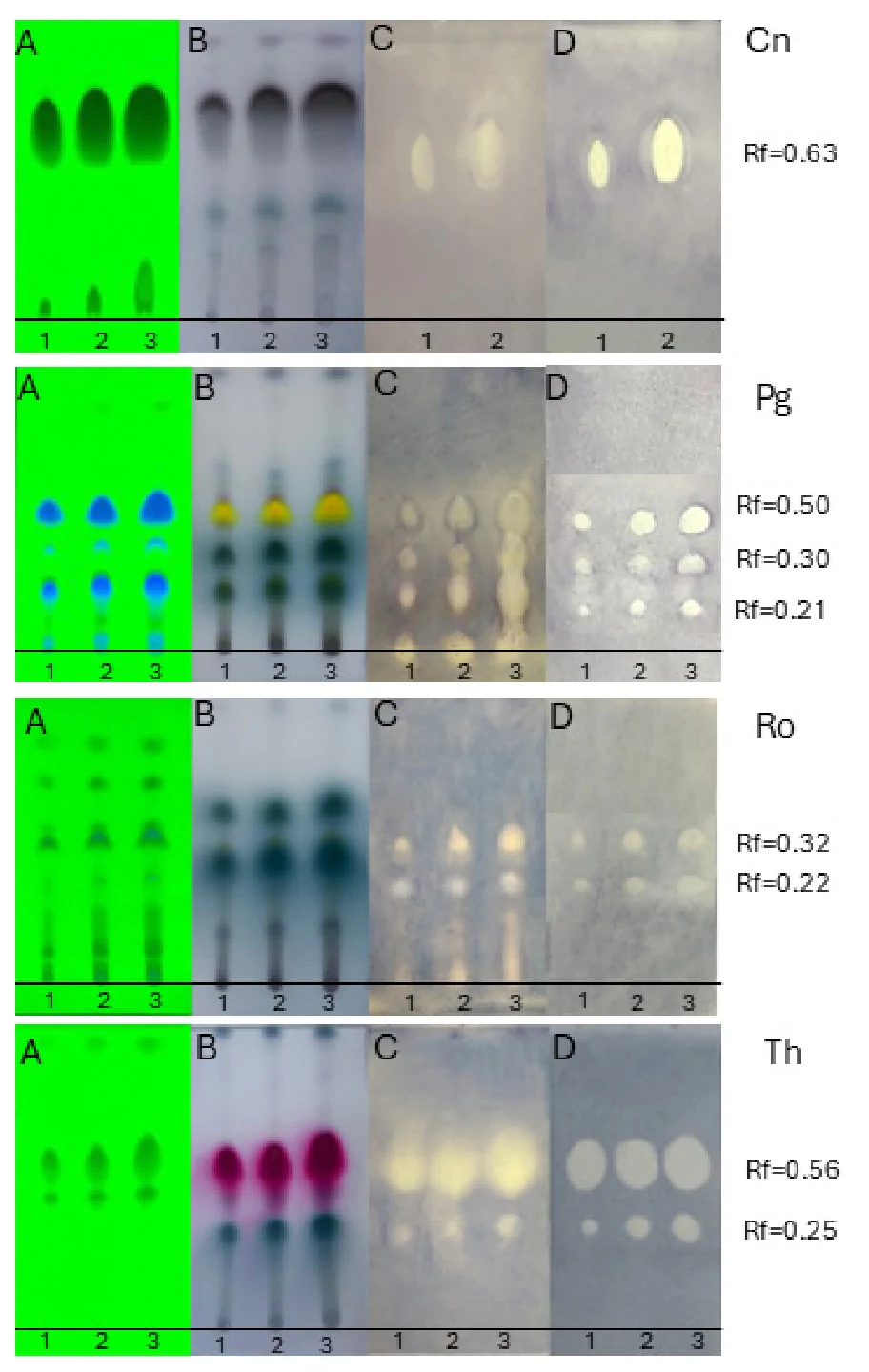
Detection of the antibacterial components of cinnamon (Cn), petitgrain (Pg), rose geranium (Ro), and thyme (Th) essential oils using Thin-Layer Chromatography-based Direct Bioautography. Order in each section: (**A**) TLC plate under UV 254 nm, (**B**) TLC plate after treatment with vanillin-sulfuric acid reagent and documented in visible light, (**C**) TLC-DB assay: Bioautograms using *P. aeruginosa* Isolate 8, (**D**) TLC-DB assay. Bioautograms using *P. aeruginosa* isolate 37 (bright zones indicate antibacterial effects). Abbreviations and concentrations: Cn: cinnamon bark EO (150 mg/mL); Pg: petitgrain EO (250 mg/mL); Ro: rose geranium EO (250 mg/mL); Th: Thyme EO (250 mg/mL). Applied volumes: 1, 2, and 3 µL for the EOs, except for cinnamon bark EO, where it was 1 and 2 µL. Mobile phases: Dichloromethane was used for cinnamon bark oil only, while toluene and ethyl acetate were applied in 93:7 (*v*/*v*) ratios. The Rf values of the active zones are indicated on the right side of the figure.

**Figure 3:**
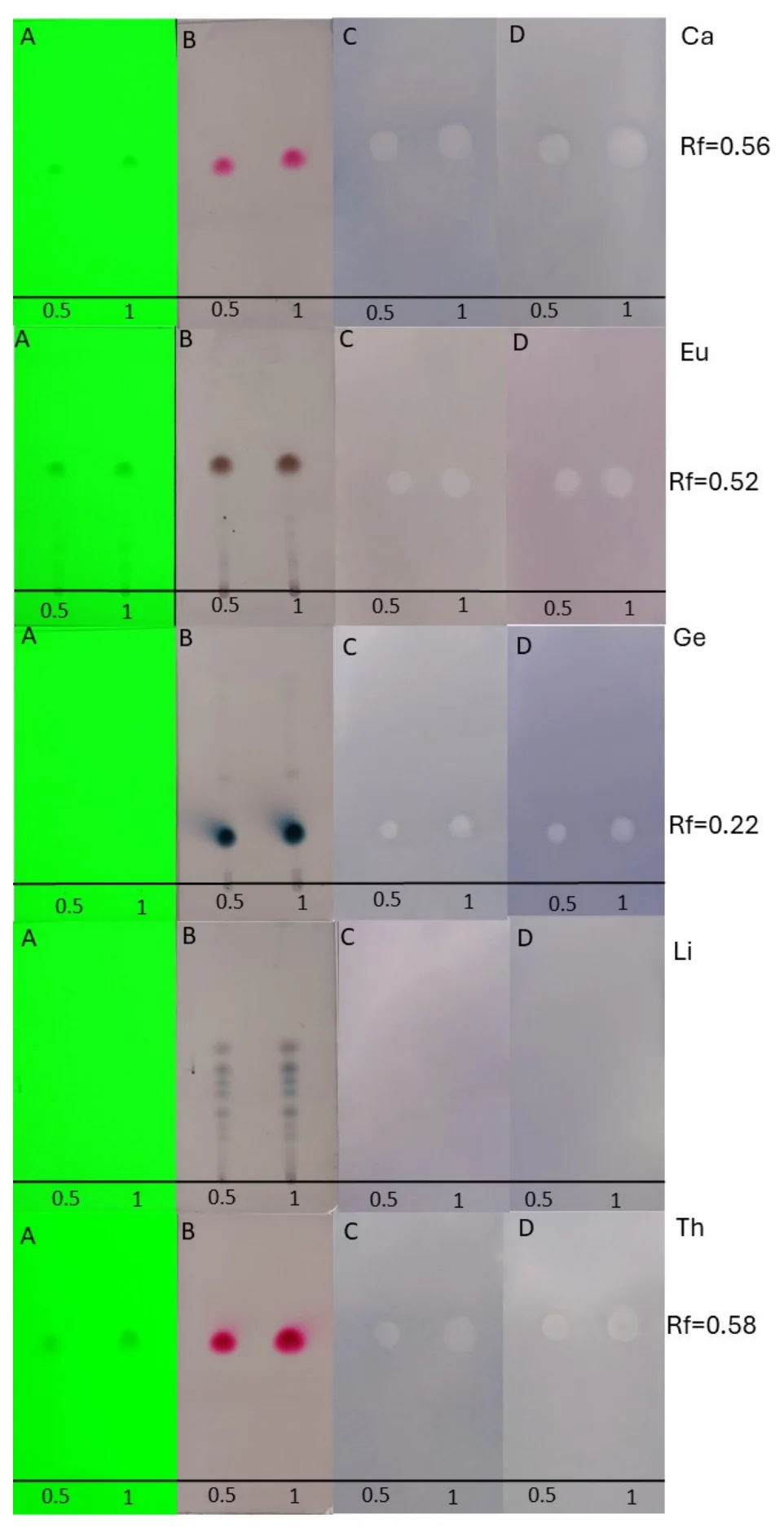
Thin-layer chromatographic separation and direct bioautography of selected essential oil constituents against two strains of *P. aeruginosa*. Reference compounds including cinnamaldehyde (Ca), eugenol (Eu), geraniol (Ge), limonene (Li), and thymol (Th) were separated on silica gel 60 F254 thin-layer chromatography (TLC) plates using toluene–ethyl acetate (95:5, *v*/*v*) as the mobile phase. Stock solutions of the compounds (20 mg/mL) were applied in volumes of 0.5 and 1 µL, corresponding to 0.01 and 0.02 mg per spot, respectively. (**A**) Chromatograms visualized under UV light at 254 nm. (**B**) Plates after vanillin–sulfuric acid derivatization for chemical visualization of separated compounds. (**C**) Direct bioautography showing antibacterial inhibition zones against the *P. aeruginosa* isolate 8. (**D**) Direct bioautography showing antibacterial inhibition zones against the *P. aeruginosa* isolate 37. The Rf values of the active zones are indicated on the right side of the figure.

**Figure 4:**
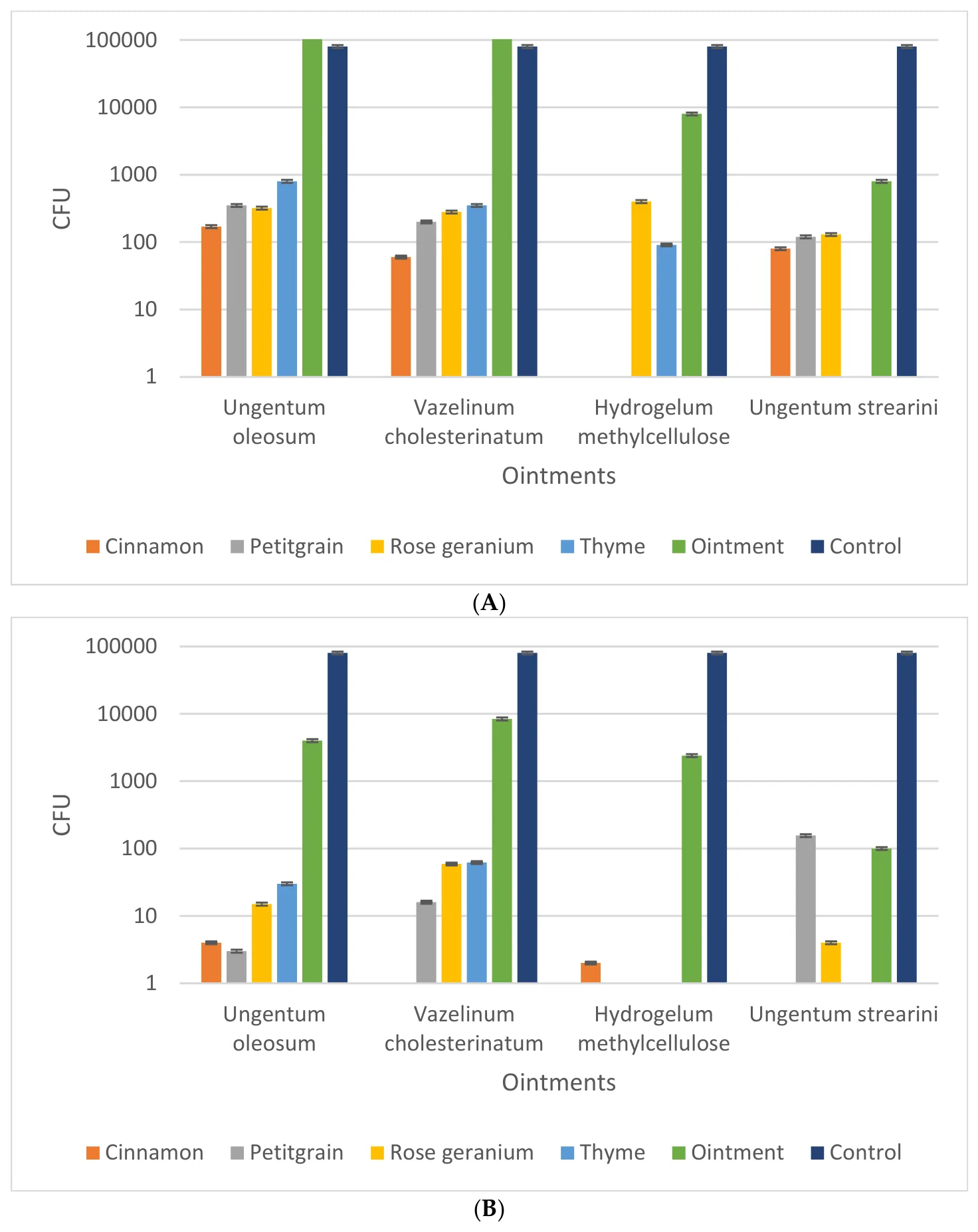
Supportive effects of the various ointment bases on the anti-*P. aeruginosa* activity of cinnamon, petitgrain, rose geranium, and thyme when applied at concentrations of 2.5 *v*/*v*% (**A**) and 5 *v*/*v*% (**B**). The results represent the number of colony-forming units after a 15-min exposure time.

**Figure 5:**
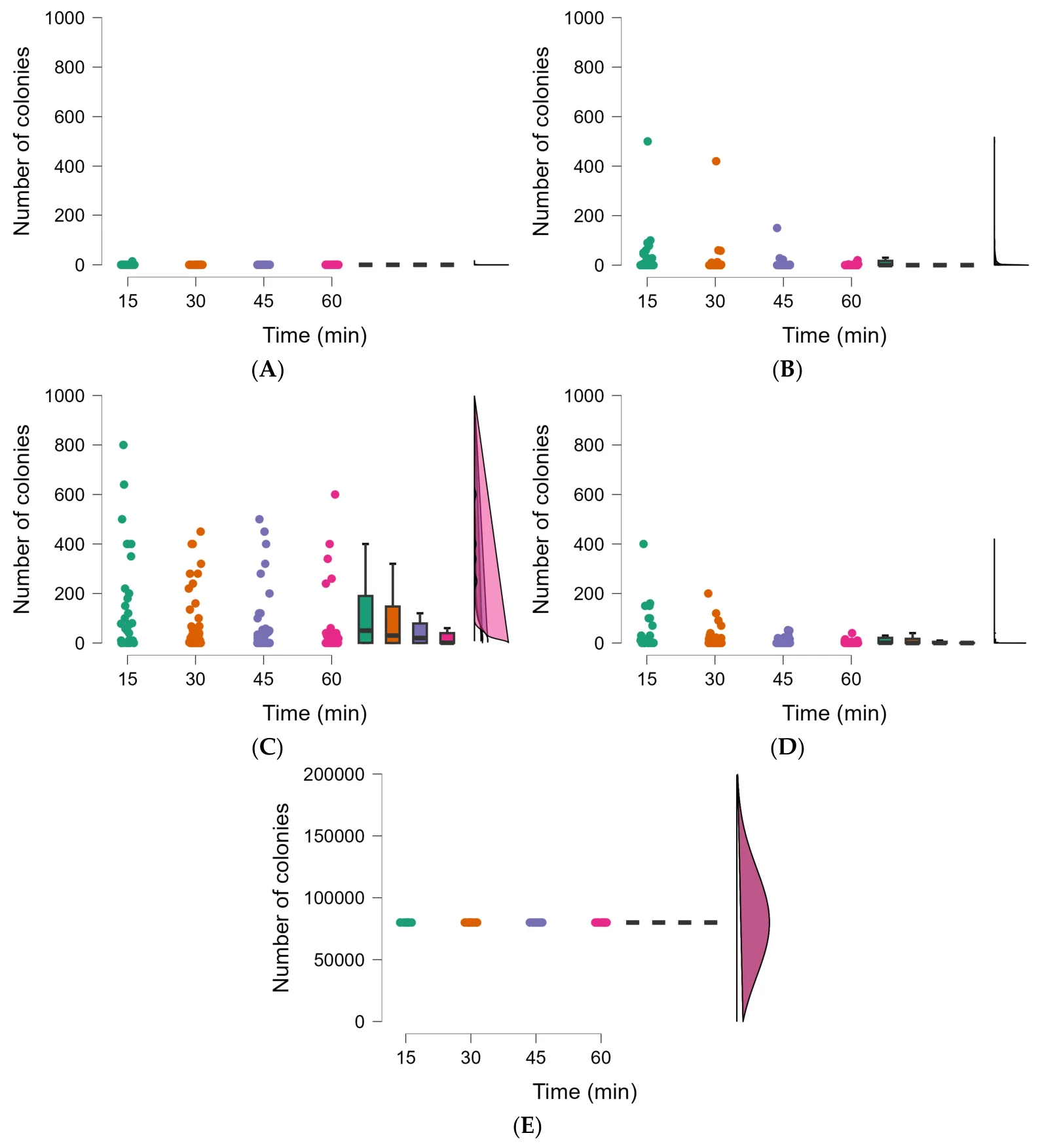
Antibacterial effects of (**A**) cinnamon, (**B**) petitgrain, (**C**) rose geranium, (**D**) thyme incorporated in methyl cellulose hydrogel as a function of time. The result for the control is (**E**). Essential oils were applied at concentrations of 2.5% and 5%, and living bacterial numbers were determined after exposure times of 15, 30, 45, and 60 min. These were then compared to the control on *P. aeruginosa* isolates 4, 8, 10, 13, 33, and 37. Raincloud plots were created using JASP 0.18.3.0.X software. Each subplot illustrates the distribution of the data, the individual data points, and the summary statistics via boxplots (the central line represents the median, the box bounds the interquartile range (IQR), and the whiskers extend to 1.5 × IQR).

**Figure 6:**
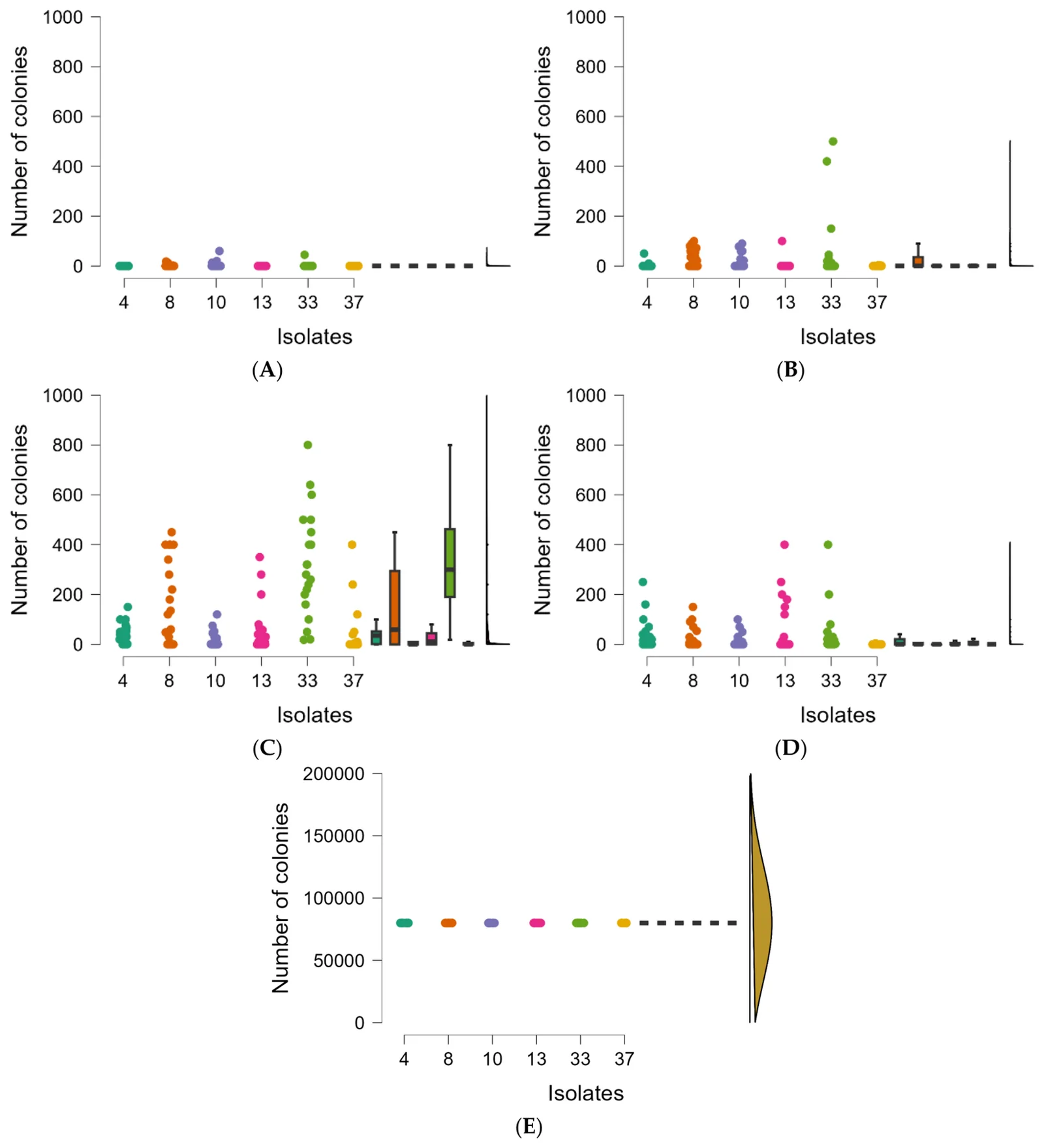
The overall cumulative effect of individual essential oils ((**A**): cinnamon, (**B**): petitgrain, (**C**): rose geranium, (**D**): thyme, and (**E**): positive control) as a function of the isolates. The raincloud plot shows germ counts measured at all time points as a single, pooled dataset. Numbers 4, 8, 10, 13, 33, and 37 represent the *P. aeruginosa* isolates used. The data were plotted using JASP 0.18.3.0. software. Each subplot illustrates the distribution of the data, the individual data points, and the summary statistics via boxplots (the central line represents the median, the box bounds the interquartile range (IQR), and whiskers extend to 1.5 × IQR).

**Figure 7:**
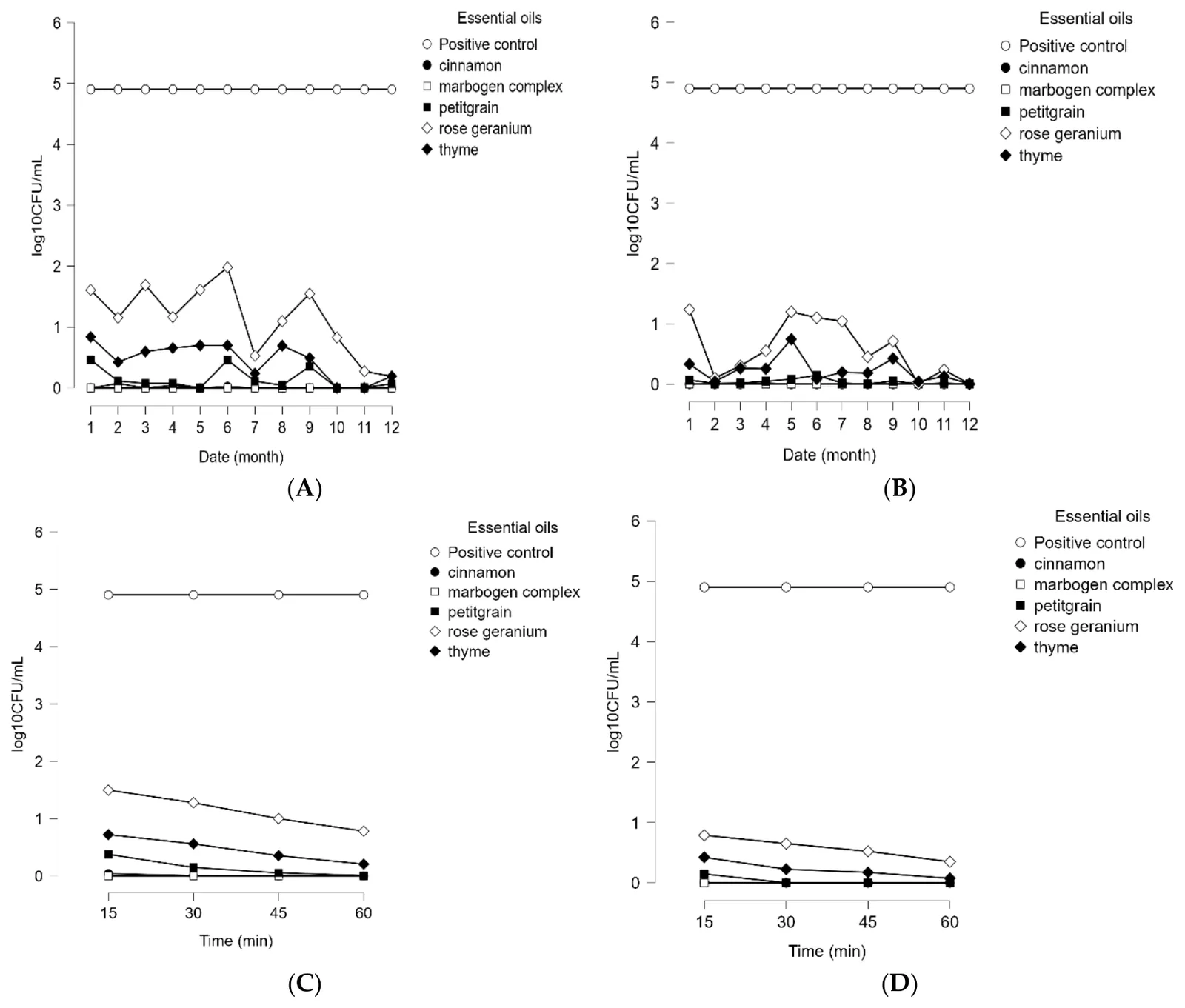
Shelf-life study of CFU over time ((**A**): isolate 8, (**B**): isolate 37) and as a function of incubation time ((**C**): isolate 8, (**D**): isolate 37).

**Table 1:** Compositions of the 4 different ointment bases, such as Normales (FoNo) Editio VIII: Hydrogelum methylcellulose FoNo VIII, *Unguentum oleosum* FoNo VIII. (Ung. oleoso), Unguentum stearini FoNo VIII. (Ung. stearin), and Vaselinum cholesterinatum FoNo VIII. (Vasel. cholest.).

Vaselinum Cholesterinatum	Unguentum Oleosum	Unguentum Stearini	Hydrogelum Methylcellulose
Alcohol cetylicus 30 g Cera alba 30 g Adeps lanae 50 g Vaselinum album 870 g Cholesterolum 20 g	Alcoholesadipislanae 50 g Cera alba 100 g Alcohol cetylicus et stearylicus 150 g Ricinus oleum virginale 700 g	Alcohol cetylicus et stearylicus 45 g Acidum stearicum 100 g Natrii laurilsulfas 5 g Solutio conservans 10 g Sorbitolum 35 g Glycerolum 85% 100 g Aqua purificata 705 g	Methylcellulosum 50 g Glycerolum 85% 100 g Solutio conservans 10 g Aqua purificata 840 g

## Data Availability

The original contributions presented in this study are included in the article. Further inquiries can be directed to the corresponding author.

## Abbreviations

The following abbreviations are used in this manuscript:

CFU	colony-forming unit
EO	essential oil
GC	gas chromatography
HM	hydrogel methylcellulose
